# The other Babinski sign in Hemifacial Spasm: a clinical marker of severity and its predictive association with η-like vA-PICA compression

**DOI:** 10.1080/07853890.2025.2596480

**Published:** 2025-12-13

**Authors:** Gang Wu, Zihao Zhang, Qingpei Hao, Zeyu Miao, Bin Li, Hu Ding, Ruen Liu

**Affiliations:** Department of Neurosurgery, Peking University People’s Hospital, Beijing, China

**Keywords:** Hemifacial spasm, the other babinski sign, posterior inferior cerebellar artery, neurovascular compression, offending vessels

## Abstract

**Objective:**

To investigate the clinical significance of the other Babinski sign in hemifacial spasm (HFS), focusing on its association with symptom severity and specific neurovascular compression (NVC) patterns.

**Methods:**

This prospective observational study evaluated 69 consecutive HFS patients undergoing microvascular decompression. The association between the other Babinski sign and HSGS scores was assessed using the chi-square test for independence, with Cramer’s V quantifying association strength. Subsequently, univariate and multivariate logistic regression analyses were performed to identify NVC patterns predictive of the other Babinski sign.

**Results:**

The other Babinski sign was present in 15/69 (21.7%) patients. Its presence was significantly associated with higher HSGS scores (*p* = 0.011), indicating greater HFS severity (Spearman’s rho = 0.355, Cramer’s *V* = 0.434, *p* = 0.003). Multivariate logistic regression identified the η-like vertebral artery-posterior inferior cerebellar artery (VA-PICA) configuration as a significant independent predictor for the other Babinski sign (Odds Ratio = 4.454, 95% CI: 1.116–17.773, *p* = 0.034).

**Conclusion:**

The other Babinski sign is a significant clinical indicator of more severe HFS and is strongly associated with the η-like VA-PICA compression pattern. This sign may aid in preoperative assessment by predicting HFS severity and specific underlying vascular NVC morphologies, potentially informing surgical strategy.

## Introduction

Hemifacial spasm (HFS) is a chronic movement disorder characterized by intermittent, involuntary contractions of the facial muscles on one side. These involuntary spasms can be triggered by routine actions like speaking or blinking and are often intensified by stress or fatigue. The resulting functional disability and significant psychosocial burden substantially impact the quality of life for affected individuals [1,2]. Epidemiologically, HFS affects 7.4–14.5 per 100,000 individuals, occurs more frequently in women, and most often presents in the fifth to seventh decades of life [3,4]. The predominant cause, accounting for over 85% of cases, is neurovascular compression (NVC) at the transitional zone (TZ) where the facial nerve exits the brainstem [5]. This region, spanning approximately 8–10 mm, is defined by the shift from central glial myelin (oligodendrocyte-derived) to peripheral Schwann cell myelin and is particularly vulnerable due to its proximity to the vertebrobasilar vasculature [6]. It has been hypothesized that chronic pulsatile compression at this site may induce a mechano-to-electrical transduction effect *via* certain mechanisms [7].

**Figure 1.**
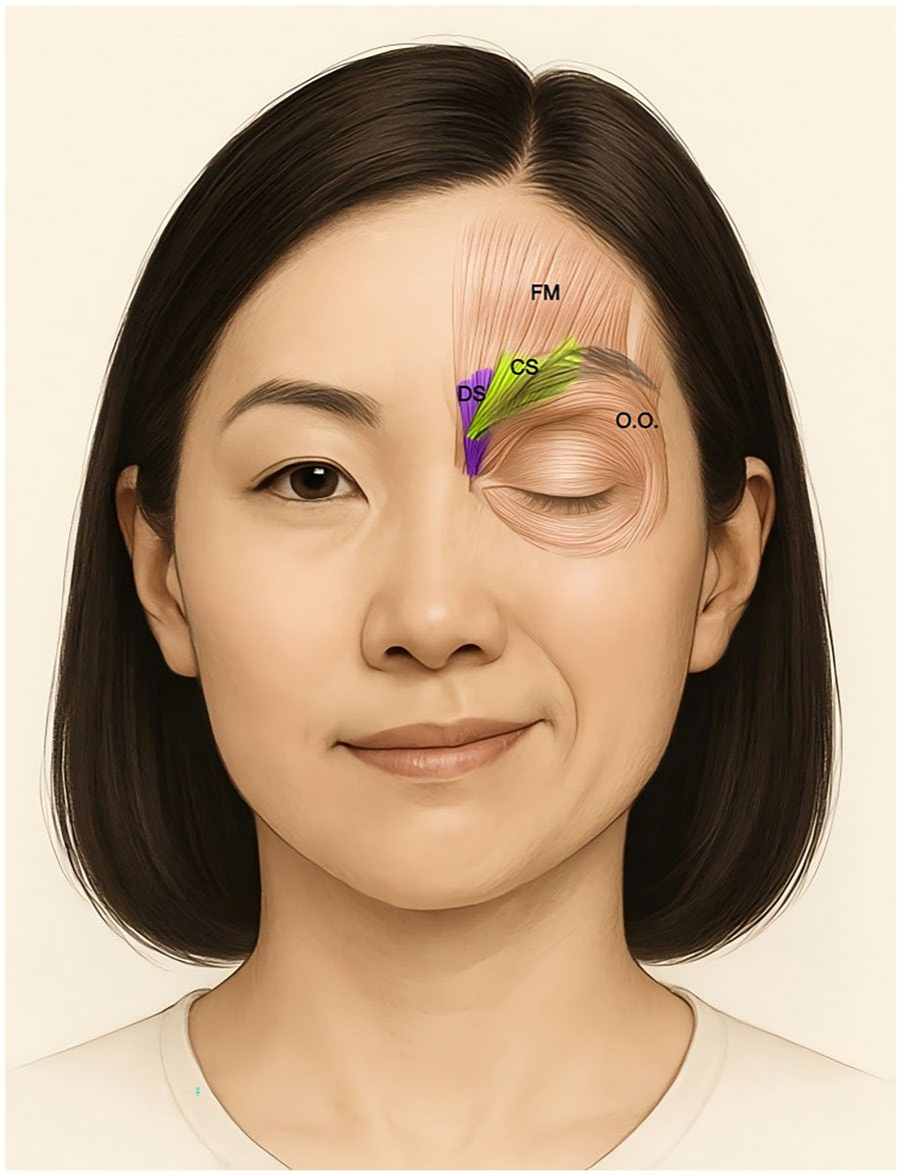
Clinical manifestation of the ‘other babinski sign’ in hemifacial spasm. Paradoxical elevation of the ipsilateral eyebrow during eye closure, characteristic of the ‘other Babinski sign’. This results from synchronous contraction of the frontalis muscle (FM) and orbicularis oculi (O.O.) muscle. FM, frontalis muscle; O.O., orbicularis oculi; DS, depressor supercilii; CS, corrugator supercilii.

In 1905, Joseph Babinski first described a distinctive phenomenon in HFS, termed the ‘other Babinski sign’ [8]. This sign manifests as synchronized contraction of the ipsilateral orbicularis oculi and frontalis muscles during voluntary eye closure, resulting in eyelid closure with paradoxical eyebrow elevation (Figure 1). Notably, the frontalis contraction may override its antagonist (orbicularis oculi), and the movement cannot be volitionally replicated [9]. Subsequent studies report a variable prevalence of this sign in HFS, ranging from 25.3% to 86%[10,11], likely influenced by methodological discrepancies such as observational techniques (direct vs.video-based) and recording duration limitations.

The ‘other Babinski sign’ serves both as a hallmark and a critical diagnostic tool of HFS. It distinguishes HFS from epileptic facial seizures or blepharospasm, where the sign is absent. This disparity suggests that the sign reflects pathophysiological mechanisms specific to primary HFS, such as facial nerve hyperexcitability secondary to NVC at the the facial nerve’s TZ at the brainstem.

Building upon this mechanistic insight, the present study aims to bridge the gap between the other Babinski sign and clinical correlates. By systematically analyzing the association between the sign and intraoperatively identified NVC patterns, we seek to address two key questions: (1) Whether the presence of the sign correlates with greater symptom severity, and (2) Whether specific compression types are more likely to elicit the sign. Clarifying these relationships is therefore crucial, as it could offer a low-cost means to identify patients with severe HFS, potentially aiding in the anticipation of underlying NVC types. Such insights would be invaluable for timely and efficient surgical planning in microvascular decompression (MVD) procedures.

## Methods

### Patient selection

The study cohort comprised 69 consecutive patients who presented with medication-refractory unilateral HFS and subsequently underwent MVD between September and November 2024.

Individuals were excluded if they had received prior treatments that could alter facial muscle responses, such as botulinum toxin therapy or previous surgical interventions. Furthermore, patients with a history of facial nerve injury (including Bell’s palsy) or significant pre-existing facial weakness (House–Brackmann grade II or higher) were not eligible. We also excluded participants with active systemic conditions, including autoimmune or neurodegenerative disorders, and those with uncontrolled comorbidities or other contraindications that posed an unacceptable risk for general anesthesia.

All study procedures adhered to the principles of the Declaration of Helsinki. The research protocol was formally approved by the Ethics Committee of Peking University People’s Hospital (Approval No. 2024PHB291-001), and every participant provided written informed consent before their inclusion in the study.

### Surgical procedure

To ensure procedural consistency, a single senior surgical team performed all MVD operations. Patients were placed in a lateral position with the head unfixed, allowing for dynamic adjustments to optimize surgical exposure. Access to the cerebellopontine angle was achieved *via* a standard 3-cm retrosigmoid craniotomy, performed with minimal cerebellar retraction.

Under microscopic magnification, the surgical corridor was developed by first dissecting the arachnoid adhesions around the glossopharyngeal, vagus, and accessory nerves (CN IX-XI). A subfloccular trajectory was then utilized to expose the facial nerve’s transitional zone. In cases where the flocculus obscured the view, the refractive properties of cerebrospinal fluid (CSF) were used to identify the neurovascular conflict accurately. The definitive decompression involved meticulously inserting Teflon felt pledgets to separate the offending vessel(s) from the nerve and brainstem. The procedure concluded with a multi-layered, watertight closure of the dura mater to prevent CSF leakage. Each operation was video-recorded in its entirety for subsequent detailed analysis.

### Definition of vascular configurations

**Figure 2.**
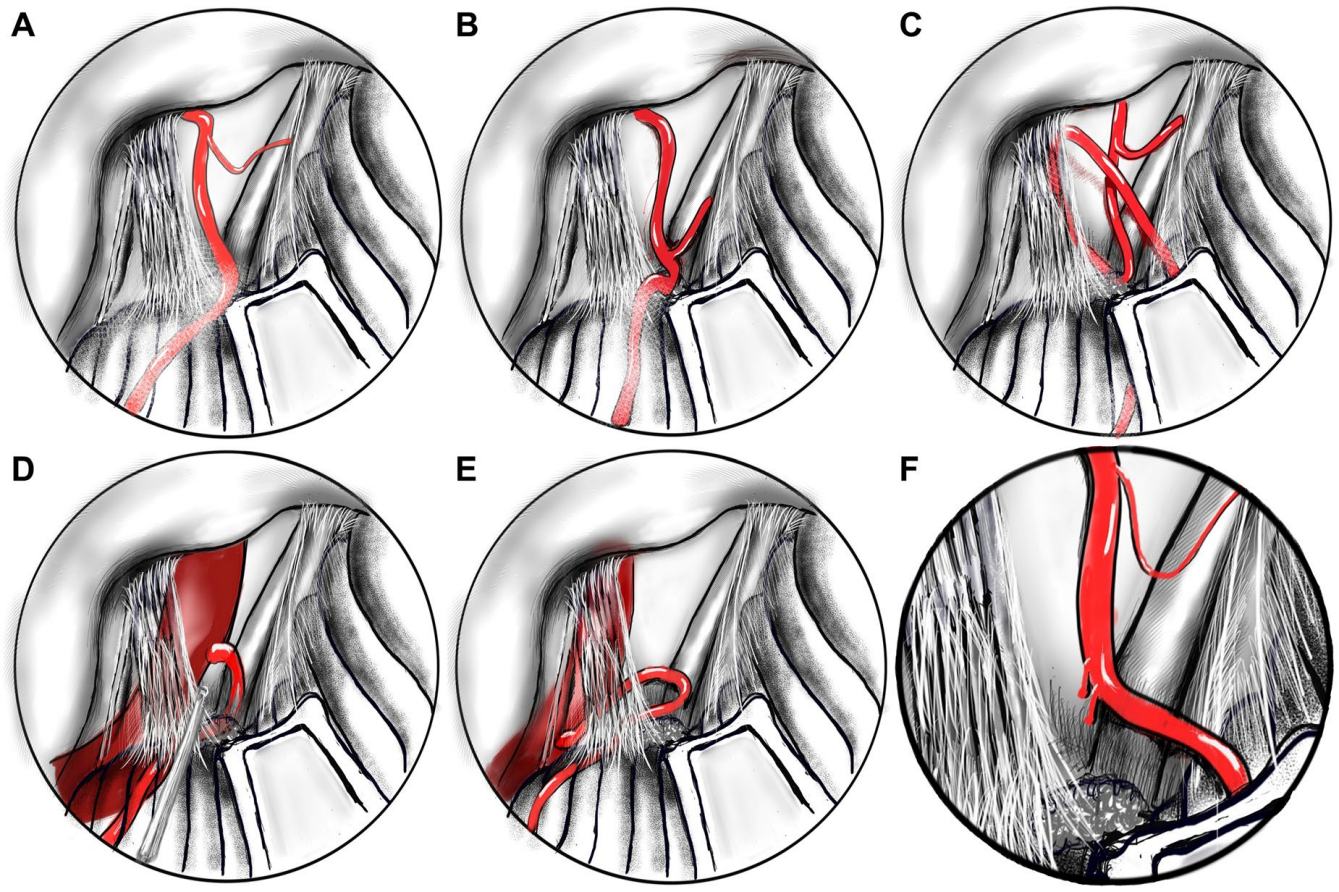
Variations in offending vessel configurations. (A) Proximal AICA bifurcation (>3 mm from REZ), categorized as AICA_REZ_distance: proximal. (B) Distal AICA bifurcation (≤3 mm from REZ), categorized as AICA_REZ_distance: distal. (C) Alpha-shaped AICA, forming a tortuous loop compressing the REZ, categorized as AICA_shape: alpha. (D) Eta-shaped VA–PICA complex, with a high-origin PICA forming a shoulder-like loop compressing the REZ, categorized as VA_PICA_shape: eta. (E) Non-eta-shaped PICA, either low-origin or non-compressive high-origin, categorized as VA_PICA_shape: non-eta. (F) Short perforating artery (≤ 2 mm in length), categorized as Perforator_length: short.

The characteristics of the offending vessels were evaluated as independent variables in this study. The location of the anterior inferior cerebellar artery (AICA) bifurcation relative to the TZ was recorded as AICA_TZ_distance, and was classified as proximal when the bifurcation point was situated more than 3 mm from the TZ **(**Figure 2A), and distal when located 3 mm or less from the TZ (Figure 2B).

The configuration of the AICA itself, termed AICA_shape, was categorized as alpha shape when the main trunk or branches of the artery formed a tortuous loop resembling the Greek letter α that compressed the TZ (Figure 2C). When this specific shape was absent—despite the presence of curved structures such as S- or C-shapes without marked tortuosity—the AICA was classified as non-alpha shape.

The spatial relationship between the vertebral artery (VA) and the TZ was assessed and denoted as VA_TZ_ relationship. This was defined as on TZ if the VA directly crossed or impinged upon the TZ, and as not on TZ if the VA did not contact or traverse this region. Similarly, the shape of the vertebral artery–posterior inferior cerebellar artery (VA–PICA) complex was described by the variable VA_PICA_shape. A classification of eta (η) shape was applied when a high-origin PICA formed a loop with a shoulder-like structure that compressed the TZ (Figure 2D). In contrast, the non-eta shape included configurations in which the PICA did not enter the TZ, originated low and affected the TZ without forming a compressive shoulder, or had a high origin but without TZ compression (Figure 2E).

Finally, perforating arteries were evaluated based on length, recorded as Perforator_length, and classified as short if ≤ 2 mm or non-short if >2 mm (Figure 2F).

### Video recording and outcome measures

To quantify the severity of HFS before surgery, a standardized video assessment protocol was implemented. To account for the fluctuating nature of HFS and to ensure consistent assessment, the evaluation was not based on passive observation alone. Patients were instructed to perform a standardized provocation maneuver (repeatedly opening and closing their eyes for at least 30 s) to reliably elicit spasms, which were then recorded and scored. Each patient’s facial movements were captured using high-definition (1080p) video for a minimum of 1.5 min. The collected video files were subsequently anonymized and randomized. An independent review was then conducted by a fellowship-trained neurosurgeon who remained blinded to all intraoperative findings and surgical details. These recordings served as the basis for the clinical scoring detailed below.

Preoperative HFS severity was quantitatively assessed using the Hemifacial Spasm Grading Scale (HSGS) **(**Table 1) [12]. This scale evaluates spasm localization, intensity, and the nature of spontaneous contractions. A score of 0 indicated no HFS manifestations. Localization was scored as 1 for isolated upper or lower facial muscle involvement (e.g. orbicularis oculi) or 2 for involvement of both. For the purpose of this scale, ‘upper facial muscles’ refers primarily to the periocular musculature (orbicularis oculi), while ‘lower facial muscles’ encompasses the perioral musculature (e.g. orbicularis oris, zygomaticus muscles). Intensity received a score of 1 for single spasms or 2 for sub-continuous spasms. Spontaneous contractions were scored as 3, increasing to 5 if contractions occurred during more than 50% of the observation period. For statistical analysis, total HSGS scores were dichotomized into mild-to-moderate (score < = 7) and severe (score > 7) HFS.

**Table 1.** The Hemifacial Spasm grading scale (HSGS).

Hemifacial Spasm	Yes - no
Localization	
Isolated upper face (e.g. orbicularis oculi)/lower face muscles	1
Involvement of both the upper and lower face muscles	2
Intensity	
Single jerks	1
Sub-continuous jerks (spasm)	2
Frequency	
Muscular contractions provoked by motor activation	1
Spontaneous contractions	
<50% the time	3
>50% the time	5

### Statistical analysis

Data were analyzed using SPSS Statistics for Windows, Version 24 (IBM Corp., Armonk, NY). Continuous variables were assessed for normality; normally distributed data are expressed as mean ± standard deviation and were compared using Student’s t-test, whereas non-normally distributed data are expressed as median (interquartile range) and were compared using the Mann-Whitney U test. Categorical variables were examined using the chi-square test or Fisher’s exact test, as appropriate.

The association between the ‘other Babinski sign’ and HSGS scores was specifically assessed using a chi-square test for independence. The strength of this relationship was quantified with the Cramer’s V coefficient, interpreted as weak (< 0.15), moderate (0.15–0.30), or strong (> 0.30). Univariate and multivariate logistic regression analyses were conducted to identify factors associated with outcomes. Variables yielding a P-value < 0.10 in the univariate analysis were included in the multivariate logistic regression model. All statistical tests were two-tailed, and a p-value <0.05 was considered statistically significant.

## Results

### Patient baseline characteristics

A total of 69 patients were included in the study and allocated into two groups based on the presence (‘Yes’ group, *n* = 15) or absence (‘No’ group, *n* = 54) of the other Babinski sign. The baseline demographic and clinical characteristics of groups are summarized in Table 2. No statistically significant differences were observed between the ‘Yes’ and ‘No’ groups regarding sex distribution, age at surgery, disease duration, or laterality of symptoms (all *p* > 0.05). However, the HSGS scores differed significantly between the groups (*p* = 0.011). Patients in the ‘Yes’ group generally presented with higher HSGS scores (Table 2).

**Table 2.** Patient demographics and clinical characteristics.

Characteristic	No (*n* = 54)	Yes (*n* = 15)	p Value
Sex, no. (%)			0.275
Male	17 (31.5%)	7 (46.7%)	
Female	37 (68.5%)	8 (53.3%)	
Age at surgery (y)	54.02 ± 9.82	58.47 ± 9.55	0.123
Disease duration (y)	4.00 (2.00–10.00)	8.00 (3.00–10.00)	0.160
Laterality			0.681
Left	32 (59.3%)	8 (53.3%)	
Right	22 (40.7%)	7 (46.7%)	
HFS Grading Scale (HSGS)			**0.010**
4	23 (42.6%)	1 (6.7%)	
5	7 (13.0%)	2 (13.3%)	
7	19 (35.2%)	6 (40.0%)	
9	5 (9.3%)	6 (40.0%)	

Data are presented as mean ± SD, median (range), or n (%). Groups are defined by the absence (No) or presence (Yes) of the other Babinski sign. P -values were calculated using Student’s t-test or Mann-Whitney U test for continuous variables and Chi-square test or Fisher’s exact test for categorical variables. A p-value < 0.05 was considered statistically significant. HFS: hemifacial spasm. The bold value indicates statistical significance (*p* < 0.05).

### Association between the other babinski sign and HSGS

To examine the relationship between the other Babinski sign and HSGS, a correlation analysis was performed. The results indicated a significant linear trend between HSGS severity grade and the other Babinski sign (linear-by-linear association test, χ ^2^=8.611, *p* = 0.003). Furthermore, Spearman’s rank correlation analysis revealed a significant positive correlation between HSGS severity grade and the other Babinski sign (rho = 0.355, *p* = 0.003). Analysis of the association strength indicated a strong association between the two variables (Cramer’s *V* = 0.434).

### Vascular predictors of the other Babinski sign

To identify potential vascular characteristics associated with the presence of the other Babinski sign, we performed logistic regression analysis. Following univariate analysis, variables demonstrating potential association (*p* < 0.10) were included in a multivariate logistic regression model.

The final multivariate model included two vascular variables: VA_TZ_ relationship and VA_PICA_shape. The overall model significantly predicted the presence of the other Babinski sign compared to the null model (Omnibus Test of Model Coefficients: χ^2^ = 8.108, *p* = 0.017). The model demonstrated good calibration according to the Hosmer–Lemeshow goodness-of-fit test (χ^2^ = 0.186, df = 2, *p* = 0.911). Among the included predictors, VA_PICA_shape was identified as a significant independent predictor of the other Babinski sign (OR = 4.454, 95% CI: 1.116–17.773, *p* = 0.034). In contrast, VA_TZ_relationship did not reach statistical significance in the multivariate model (OR = 3.093, 95% CI: 0.722–13.258, *p* = 0.128), although a trend toward increased odds was observed.

## Discussion

The other Babinski sign, a paradoxical elevation of the eyebrow during voluntary eye closure, has long been recognized as a distinctive clinical feature in HFS [9–11]. However, its precise clinical implications and relationship with underlying NVC characteristics have remained areas requiring further elucidation. This study aimed to bridge this gap by investigating the associations between the other Babinski sign, HFS severity, and specific patterns of offending vessel compression. Our findings establish two key points: first, the presence of the sign is a significant indicator of greater HFS severity, as demonstrated by its strong positive correlation with higher HSGS scores. Second, a specific vascular compression pattern—the η-like VA-PICA shape—is independently associated with the likelihood of observing this sign. These results underscore the clinical utility of the other Babinski sign beyond its diagnostic role, suggesting its potential as a marker for both symptom severity and particular NVC patterns.

The association between the other Babinski sign and greater HFS severity is best understood from a biomechanical perspective, centered on the unique antagonistic interplay of the frontalis muscle. The frontalis is the sole elevator of the eyebrow, and it acts in constant opposition to a powerful group of depressor muscles, including the orbicularis oculi and the glabellar complex (e.g. depressor supercilii, the oblique and transverse portions of the corrugator supercilii) [13–15]. We postulate that in milder HFS, aberrant nerve discharges primarily affect more easily activated facial muscles, and any minor co-contraction of the frontalis is effectively counteracted by its powerful antagonists, remaining subclinical. However, as the disease progresses in severity, the involuntary contractions of the orbicularis oculi are met by an equally spasmodic and powerful contraction of the entire frontalis muscle [16]. This intense antagonistic struggle results in the paradoxical eyebrow elevation characteristic of the sign. Therefore, the presence of the sign represents a physical manifestation of a more widespread and intense neuromuscular conflict, which logically correlates with the higher HSGS scores observed in our study.

The pathophysiological basis for this association between the sign and increased severity may lie in the hyperexcitability of the facial motor nucleus. As some researchers have proposed, this distinctive phenomenon may be related to differences in the excitability thresholds between the motor neuron pools of the frontalis and orbicularis oculi muscles [11]. Specifically, the motor neurons innervating the frontalis muscle may be fewer in number or possess higher activation thresholds, making them less likely to be recruited under physiological conditions. However, in patients with HFS, aberrant discharges from the facial nucleus may synchronously activate these otherwise less excitable neurons, resulting in abnormal contraction of the frontalis muscle. This supports a reverse inference, consistent with our findings: the presence of the other Babinski sign may indicate a higher level of facial motor neuron excitability and thus a more severe disease state.

Our finding that the η-like VA-PICA shape is a strong predictor for the sign suggests a potential anatomical correlate for this heightened neurovascular conflict. Studies have shown that the PICA is the second most common offending vessel following the AICA [17,18]. Specifically, a high-origin PICA, often referred to as the η-like VA-PICA configuration, a more aggressive compression pattern [19]. Its superior origin allows it to readily cross the TZ, often becoming embedded laterally within the pontomedullary sulcus and thereby tightly compressing this zone.Typically, these high-origin PICAs are characterized by a larger diameter and greater pulsation amplitude. Moreover, the short recurrent loop of such a PICA can directly transmit vascular tension from the vertebral artery to the TZ, imposing greater pressure on the facial nerve [20]. This unique anatomical structure, therefore, creates the specific high-tension conditions that provide a physical basis for the profound neuropathological changes discussed next.

This link between a specific, severe vascular compression pattern and a clinical sign of high disease severity points towards a unified pathophysiological model centered on neuronal hyperexcitability. We hypothesize that the intense and sustained mechanical stress can induce a widespread piezoelectric effect, characterized by increased quantal inward currents that persistently elevate facial nerve nucleus excitability [21–23]. Indeed, our previous investigation in this same patient cohort demonstrated that fluctuations in central excitability can be clinically captured using the glabella tap reflex, providing evidence for this underlying dynamic state [24]. Such generalized and sustained hyperexcitability of the facial nerve nucleus, in turn, contributes to more severe HFS symptoms. This increased severity, in turn, suggests that significant frontalis muscle involvement manifests as the other Babinski sign. Within this hypothetical model, the η-like PICA could represent a potent anatomical ‘cause’ that may lead to the neurophysiological ‘effect’ of widespread hyperexcitability, potentially culminating in the clinical ‘manifestation’ of the other Babinski sign. This elegantly explains why the sign serves as a marker for both a more severe disease state and a specific underlying vascular morphology.

Consequently, our findings suggest that the other Babinski sign may hold significant auxiliary value in clinical practice. As a low-cost, non-invasive clinical marker, its presence could preoperatively alert surgeons to a higher probability of encountering a complex, high-tension NVC pattern, such as an embedded η-like PICA. If validated by larger, prospective studies, this foresight could prove helpful for surgical planning in MVD procedures, perhaps allowing for better anticipation of certain surgical challenges.

This study has several limitations. First, the modest sample size (*n* = 69) may have restricted our statistical power to detect more subtle associations and our multivariate model is susceptible to overfitting, and the wide 95% confidence interval for the odds ratio reflects a lack of precision. Relatedly, our classification of vascular compression was focused on the η-like PICA configuration; other complex patterns, such as multi-vessel or branching artery compression, were not analyzed as distinct subgroups due to their low prevalence in our cohort, and likely represent another important cause of high-tension neurovascular conflict. Furthermore, inherent inter-individual variability in natural facial expressions and frontalis muscle anatomy might have influenced the manifestation of the other Babinski sign, potentially introducing measurement variability. Variations in vessel diameter and pulsation characteristics, even with high-origin PICAs, could also alter the compressive effect. While the HSGS is a relatively objective tool, an element of subjectivity can remain when scoring subtle movements. Furthermore, we acknowledge that any categorized scale has inherent limitations in capturing the full, continuous spectrum of a fluctuating neurological disorder. Lastly, our hypotheses concerning underlying mechanisms are based on clinical and anatomical observations and await direct corroboration from real-time neurophysiological monitoring. Consequently, future prospective studies with larger cohorts are warranted to validate these findings and more deeply investigate these complex relationships.

## Conclusion

Our findings highlight the other Babinski sign as a clinically relevant indicator in HFS, extending beyond its diagnostic utility. We established its strong association with increased HFS severity (HSGS scores) and a higher likelihood of an underlying η-like VA-PICA compression. This suggests the other Babinski sign might serve as a preliminary indicator in preoperative assessment, raising the possibility of greater disease severity and certain NVC patterns, although further validation is warranted to solidify its role in routine clinical practice.

## Data Availability

The data that support the findings of this study are available from the corresponding author upon reasonable request.
